# Cuproptosis-associated CDKN2A is targeted by plicamycin to regulate the microenvironment in patients with head and neck squamous cell carcinoma

**DOI:** 10.3389/fgene.2022.1036408

**Published:** 2023-01-09

**Authors:** Kaihui Fan, Yuke Dong, Tao Li, Yujie Li

**Affiliations:** Department of Otorhinolaryngology, Head and Neck Surgery, Zhengzhou Central Hospital affiliated with Zhengzhou University, Zhengzhou, Henan, China

**Keywords:** CDKN2A, head and neck squamous cell carcinoma (HNSCC), Cuproptosis, systemic homeostasis, multiomics

## Abstract

Head and neck squamous cell carcinoma (HNSCC), the most common malignancy of the head and neck, has an overall 5-year survival rate of <50%. Genes associated with cuproptosis, a newly identified copper-dependent form of cell death, are aberrantly expressed in various tumours. However, their role in HNSCC remains unknown. In this study, bioinformatic analysis revealed that the cuproptosis-related gene CDKN2A was correlated with the malignant behaviour of HNSCC. Kaplan-Meier (KM) curves showed that patients with high CDKN2A expression had a better prognosis. Multiomic analysis revealed that CDKN2A may be associated with cell cycle and immune cell infiltration in the tumour microenvironment and is important for maintaining systemic homeostasis in the body. Furthermore, molecular docking and molecular dynamics simulations suggested strong binding between plicamycin and CDKN2A. And plicamycin inhibits the progression of HNSCC in cellular assays. In conclusion, this study elucidated a potential mechanism of action of the cuproptosis-associated gene CDKN2A in HNSCC and revealed that plicamycin targets CDKN2A to improve the prognosis of patients.

## Background

Head and neck squamous cell carcinoma (HNSCC) is the most common malignant tumour of the head and neck that develops in the mucosal epithelium of the mouth, pharynx, larynx, nasal cavity, and sinus cavities (Taberna et al., 2017). It is the sixth most common malignancy worldwide, with approximately 890,000 new cases and 450,000 deaths owing to HNSCC reported worldwide in 2018 (Bray et al., 2018; Ferlay et al., 2019). Although significant progress has been made in the combined use of surgery, radiotherapy, chemotherapy and targeted therapy for the treatment of HNSCC, 40%–50% of patients have post-treatment relapse, and the overall 5-year survival rate is <50% (Canning et al., 2019). Therefore, biomarkers that can improve HNSCC prognosis need to be identified.

Tsvetkov et al., (2022) recently described a new copper-dependent type of cell death, cuproptosis, closely related to mitochondrial respiration. Copper binds to fatty acylated proteins in the tricarboxylic acid cycle during mitochondrial respiration, resulting in the aggregation of fatty acylated proteins and low expression of iron–sulfur cluster proteins, thereby inducing proteotoxic stress and eventually leading to cell death (Tsvetkov et al., 2022). There have been four genes identified for cuproptosis that are positively regulated (*FDX1*, *LIAS*, *LIPT1*, *DLD*, *DLAT*, *PDHA1*, *PDHB* and *ATP7B*) and eight genes that are negatively regulated (*MTF1*, *GLS*, cyclin-dependent kinase inhibitor 2A [*CDKN2A*] and *SLC31A1*) (Tsvetkov et al., 2022). Biological processes such as tumour cell proliferation, vascular growth, and metastasis have been shown to be significantly affected by copper, an essential element for mitochondrial respiration and iron uptake (Ruiz et al., 2021; Oliveri, 2022). There are a number of factors that regulate and maintain the body’s intake, excretion, and metabolism of copper. Copper-induced cell death or abnormal copper metabolism can occur when copper homeostasis is disrupted in the body. Previous research reported that the cuproptosis-related gene *LIPT1* is significantly correlated with prognosis and immune infiltration in melanoma (Lv et al., 2022). Similar findings have been reported by Yun Y et al. indicating that SLC31A1, DX1 and TP7B0 are associated with lung cancer (Yun et al., 2022). Several studies have shown that cuproptosis-related genes are associated with poor prognosis, reduced drug sensitivity, and tumor microenvironment in renal clear cell carcinoma (Ji et al., 2022). However, further studies are needed to determine the effects of cuproptosis on HSNCC.

Bioinformatic tools have become increasingly popular with the rapid development of high-throughput technology for assessing prognostic markers and studying mechanisms (Wei et al., 2019; Shi et al., 2021; Luo et al., 2022a; Luo et al., 2022b; Chen et al., 2022; Lin et al., 2022; Mei et al., 2022; Xuan et al., 2022; Zhao and Jiang, 2022). In melanoma, lung cancer, and renal clear cell carcinoma, cuproptosis-related genes have been studied. In recent studies, researchers examined ten cuproptosis-related lncRNAs associated with immune function and prognosis in HNSCC (Li et al., 2022). However, the functions and mechanisms of action of cuproptosis-related genes in HNSCC warrant further investigation. Molecular dynamics simulation serves as one of the important tools for assessing the stability of drug-targeting ligands for the development of oncological therapeutics (Chikan and Vipperla, 2015; Thai et al., 2015). The aim of this study was to identify cuproptosis-related genes associated with poor prognosis of HNSCC and screen for potential candidate drugs for its treatment using molecular docking and molecular dynamics simulations.

## Materials and methods

### Gene identification and data acquisition

TCGA-HNSCC data were analyzed to identify differentially expressed genes (DEGs) between tumours and healthy tissues as previous researches (Tomczak et al., 2015; Kang et al., 2021). Based on the intersection of tumour-associated DEGs and cuproptosis-associated genes, *CDKN2A* was identified as a key gene. We investigated the correlation between *CDKN2A* expression and clinical staging of HNSCC. *CDKN2A* prognostic significance was evaluated using KM curves. In order to investigate the performance of CDKN2A for predicting 1, 3 and 5 year overall survival (OS), ROC curves were plotted using the time ROC package. Furthermore, ROC curves were used to assess the relationship between *CDKN2A* and HNSCC clinical characteristics.

### CDKN2A function assessment

For further analysis, RNA-seq data were extracted from TCGA, and log2-transformed gene expression data were obtained (Tomczak et al., 2015). Based on the median CDKN2A expression, tumour samples were divided into high and low-expression groups for survival analysis. In the high and low CNKN2A expression groups, DEGs were screened using the limma R package. Adjusted *p*-values of <.05 and |logFC| values of >1 were used as the screening criteria for significant DEGs (Ritchie et al., 2015). The ggplot2 package was used to plot volcano and heat maps to visualise the expression of 17 significant DEGs. Data on CDKN2A mutations was downloaded from TCGAbiolinks and visualized using track Viewer (Colaprico et al., 2016; Ou and Zhu, 2019).

### Gene set variation analysis (GSVA)

The “GSVA” package was used to analyze all CDKN2A-associated DEGs, followed by the “limma” package to identify high and low CDKN2A expression levels (Hänzelmann et al., 2013; Lin et al., 2021).

### GSEA and KEGG enrichment analysis

GSEA was performed on DEGs associated with *CDKN2A*, KEGG enrichment analysis was performed with gseKEGG, and pathways of interest were visualized using ggplot2 (Subramanian et al., 2005; Ito and Murphy, 2013).

### Immuno-infiltration analysis

In order to calculate 28 immune cells, ssGSEA was used on gene expression profile data (Barbie et al., 2009). Based on *CDKN2A* expression levels, 28 immune cells were compared between groups with high and low expression levels of the protein. The Pearson correlation coefficient was used to examine the correlation between CDKN2A and immune cell infiltration further. Pearson correlation coefficient was used to analyze the correlation between each type of immune cell.

### Acquisition and optimization of FDA structures

Food and Drug Administration (FDA) approved 2,568 small molecules (as of 2022-01-04). We downloaded the small molecule structures from Drug Bank (http://www.drugbank.com/) in SDF format (Luo et al., 2022b). In RDKit, the Experimental-Torsion Basic Knowledge Distance Geometry (ETKDG) algorithm was used to generate 3D conformations based on the modified distance geometry algorithm, while the MMFF94 stand was used to optimize small molecule structure and energy using the MMFFOptimize Molecule module (Li et al., 2022).

The structures of the proteins were obtained from the Uniport website (https://www.uniprot.org/). PDB structure 1A5E obtained under CDKN2A entry (P42771) using gene name query. Docking was carried out using Smina Chikan and Vipperla, (2015). Protein-Ligand Interaction Profiler (Plip, https://plip-tool.biotec.tu-dresden.de/plip-web/plip) was used to analyze docking results.

### Analysis of PPI networks and gene networks

By using the STRING website and cytoscape software, we explored CDKN2A’s protein-protein interaction network (Shannon et al., 2003; Szklarczyk et al., 2021). Genemania was used to analyze the *CDKN2A* network (Warde-Farley et al., 2010).

### Construction of ceRNA network

Firstly, the multiMiR package was used to find miRNAs related to *CDKN2A* (Huang et al., 2019a). The LncRNADisease database was then used to identify HNSCC-related LncRNAs, and the miRTarBase database was used to identify shared miRNAs with HNSCC-related LncRNAs (Bao et al., 2019; Huang et al., 2019b). Cytoscape is used for the final visualization (Shannon et al., 2003).

### Drug analysis

To identify drugs that may act on DEGs (99 highly expressed genes and 56 lowly expressed genes) between high and low expression groups of CDKN2A, the cmap website was accessed (http://clue.io/).

### Molecular dynamics simulation (MDS)

The lowest energy conformation was selected as the kinetic initial conformation. The quantitative software Orca was used to perform quantum chemical optimization for small molecules under B3LYP/6-31G* basis set conditions, involving corrections for bond lengths, bond angles, dihedral angles, and calculations of RESP2 at 0 fixed charges. Gromacs 2019.6 was chosen as the kinetic simulation software, amber14sb was chosen as the protein force field, and Gaff2 force field was chosen for small molecules, and the TIP3P water model was used to add TIP3P water model to the complex system to build a water box and add sodium ions to equilibrate the system. Under elastic simulation by Verlet and cg algorithms, Particle-mesh Ewald (PME) deals with electrostatic interactions using the steepest descent method for energy minimization for the maximum number of steps (50,000 steps). The Coulomb force cutoff distance and van der Waals radius cutoff distance were both 1.4 nm. Finally the system was equilibrated with the regular system (NVT) and isothermal isobaric system (NPT), and the MD simulations were performed at room temperature and pressure for 100 ns. During the MD simulation, the hydrogen bonds involved were constrained using the LINCS algorithm with an integration step of 2 fs. The PME method was calculated with a cutoff value set to 1.2 nm and a non-bond interaction cutoff value set to 10 Å. The V-rescale temperature coupling method was used to control the simulation temperature at 300 K and the Berendsen method to control the pressure at 1 bar. Additionally, 30 ps of NVT and NPT equilibrium simulations were performed at 300 K. Finally, 50 ns of finished MD simulations were performed for the protein–ligand complex system. Root mean square fluctuations (RMSF) were used to observe the local loci variation structure of the system during the simulation (the fluctuation cutoff was set to 0.2). The radius of gyration (Rg) was used to evaluate the tightness of the system structure. The RMSF can observe the local loci variation of the system during the simulation.

### Calculation of free energy of binding of proteins and small molecules

The MD trajectory operation is calculated by the following equation:

$${\Delta G}_{bind}={\Delta G}_{complex}–\;\left({\Delta G}_{receptor}+{\Delta G}_{ligand}\;\right)$$

$${=\Delta E}_{internal}+{\Delta E}_{VDW}+{\Delta E}_{elec}+{\Delta G}_{GB}+{\Delta G}_{SA}$$

In the above equation, ${\Delta E}_{internal}$ represents internal energy, ${\Delta E}_{VDW}$ represents van der Waals interaction, and ${\Delta E}_{elec}$ represents electrostatic interaction. Internal energy includes bond energy (Ebond), angular energy (Eangle), and torsion energy (Etorsion). ${\Delta G}_{GB}$ and ${\Delta G}_{SA}$ are collectively referred to as the solvation free energy. $G_{GB}$ is the polar solvation free energy and $G_{SA}$ is the non-polar solvation free energy. We used the GB model for calculating ${\Delta G}_{GB}$ (Weiser et al., 1999). The ${\Delta G}_{SA}$ was calculated based on the product of surface tension (*γ*) and solvent accessible surface area (SA): ${\Delta G}_{SA}=0.0072\times \Delta SA$ . The entropy variation was neglected in this study due to the high consumption of computational resources with low accuracy.

### Cell culture and wound-healing assay

Human laryngeal cancer cell lines (TU212) were obtained from the Cell Bank of the Chinese Academy of Sciences. For cell culture, Dulbecco’s modified Eagle’s medium (DMEM) containing 10% fetal bovine serum (FBS) was used. In an incubator with 5% CO2 and 37°C, 10%FBS and 1% penicillin all cell lines were cultured. In TU212 cells, plicamycin was applied for 14 days at 10 nmol/L. Incubation at 37°C was carried out for both treated and untreated cells plated on 10-cm culture dishes. With a plastic pipette tip, a lane was scratched through the confluent monolayers, followed by addition of DMEM, 1% FBS, and 10 nmol/L plicamycin. Several wounded areas were observed and then photographed through a microscope 24 h after the scratch.

### Statistical analysis

Standard error of the mean is indicated by bars on figures, and was calculated using Microsoft Office Excel 2016. All experiments were performed with at a minimum of triplicate samples, and all *p*-values were calculated with two-tailed t-tests.

## Results

### Data acquisition and screening of DEGs

**FIGURE 1:**
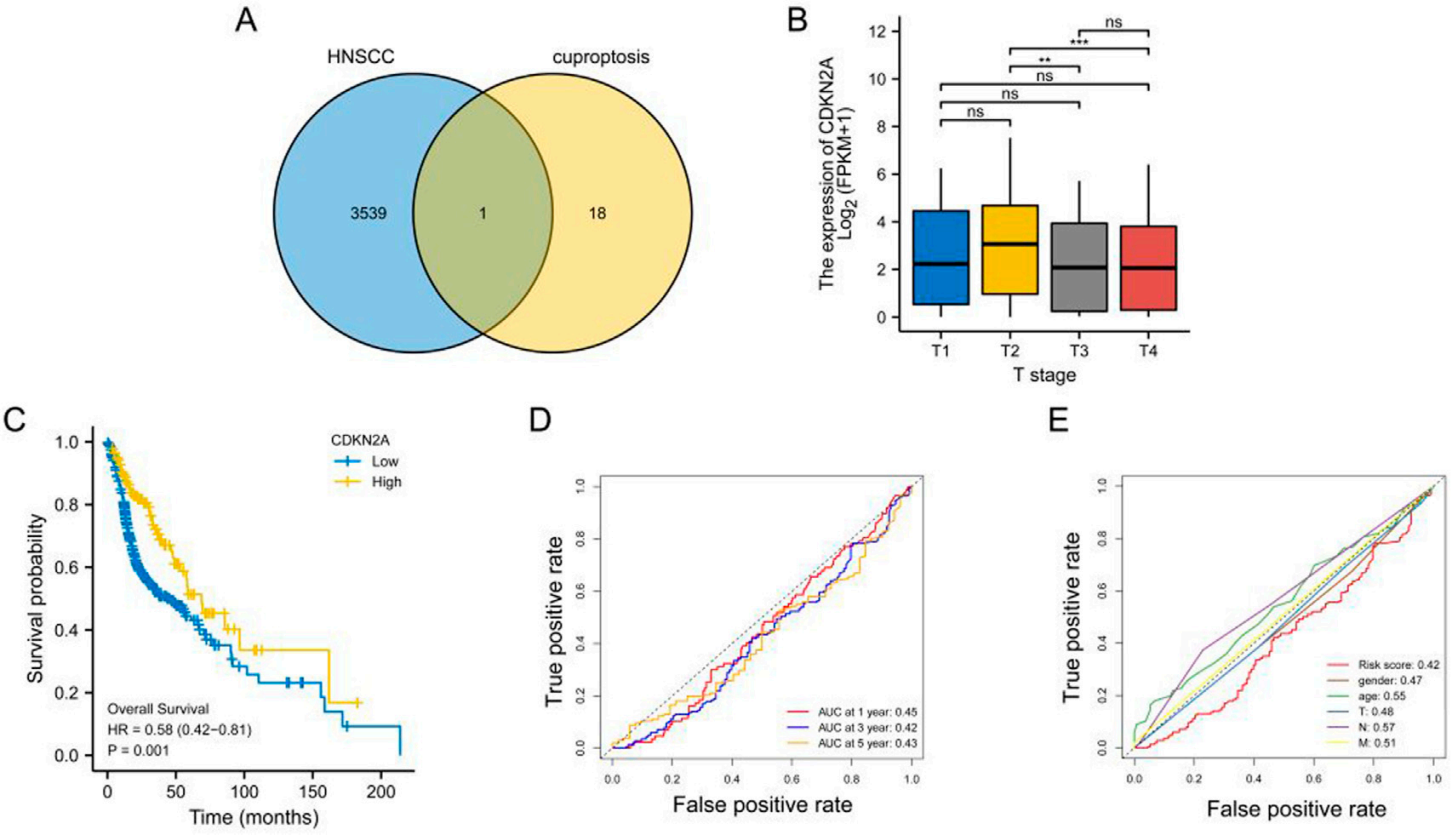
Identification of key genes. **(A)** Venn diagram demonstrating the results of intersection analysis of differentially expressed genes and cuproptosis-related genes; **(B)** Bar graph demonstrating the difference in CDKN2A expression among patients with different T stages; **(C)** KM curve demonstrating a better prognosis for patients with head and neck squamous cell carcinoma with high CDKN2A expression; **(D)** ROC curve demonstrating the predictive performance of CDKN2A for 1-, 3- and 5-year OS; **(E)** ROC curves demonstrating the relationship between clinical characteristics and the predictive performance of CDKN2A for clinical prognosis.

After intersecting tumour-associated DEGs with cuproptosis-related genes, CDKN2A was identified as a key gene (Figure 1A), which may play a critical role in tumour development through cuproptosis. CDKN2A expression was significantly higher among patients with T2-stage HNSCC than among patients with T3-and T4-stage HNSCC (Figure 1B), indicating that CDKN2A expression is high in early malignant tumours and low in advanced malignant tumours. KM curves demonstrated that high CDKN2A expression was associated with a better clinical prognosis (Figure 1C). The area under the ROC curves indicated a more significant protective effect of CDKN2A on 1-, 3- and 5-year OS (Figure 1D). In addition, age; sex; T, N, and M stages and CDKN2A were found to have predictive significance for the prognosis of HNSCC (Figure 1E).

### Characterisation of CDKN2A expression and enrichment analysis

**FIGURE 2:**
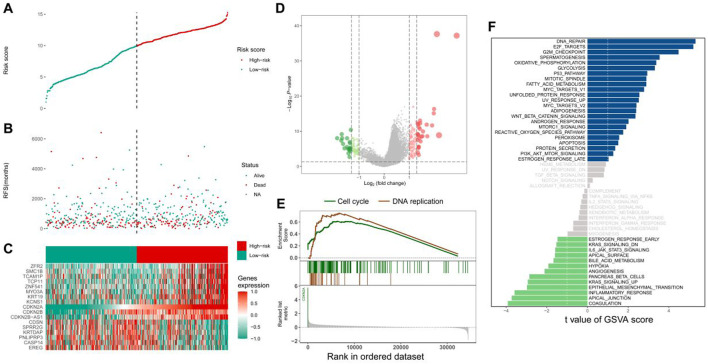
Relevant functions of CDKN2A. **(A)** Tumour samples in TCGA database were divided into the high- and low-expression groups according to median CDKN2A expression; **(B)** Scatter plot demonstrating the difference in survival status between the two groups; **(C)** Top 17 differentially expressed genes; **(D)** Volcano plot demonstrating the distribution of differentially expressed genes; **(E)** GSEA; **(F)** Bar graph demonstrating the differentially enriched pathways between the two groups.

HNSCC samples in TCGA cohort were divided into the high- and low-CDKN2A-expression groups based on the median CDKN2A expression. The scatter plot demonstrated differences in the survival status of patients between the two groups (Figures 2A, B). A total of 155 DEGs were identified in the two groups; of which, 99 were upregulated and 56 were downregulated (Figure2D). The top 17 genes with the most significant differences in expression were visualised on a heat map (Figure 2C). GSEA suggested that these genes were significantly enriched in pathways associated with cell cycle and DNA replication in the high-CDKN2A-expression group (Figure 2E). In addition, GSVA suggested that the genes were enriched in pathways associated with DNA repair, E2F targets and the G2M checkpoint in the high-CDKN2A-expression group and pathways associated with coagulation, apical junction and inflammatory response in the low-CDKN2A-expression group (Figure 2F). These results suggest that high CDKN2A expression is associated with activation of the cell cycle in HNSCC.

### Immune infiltration analysis

**FIGURE 3:**
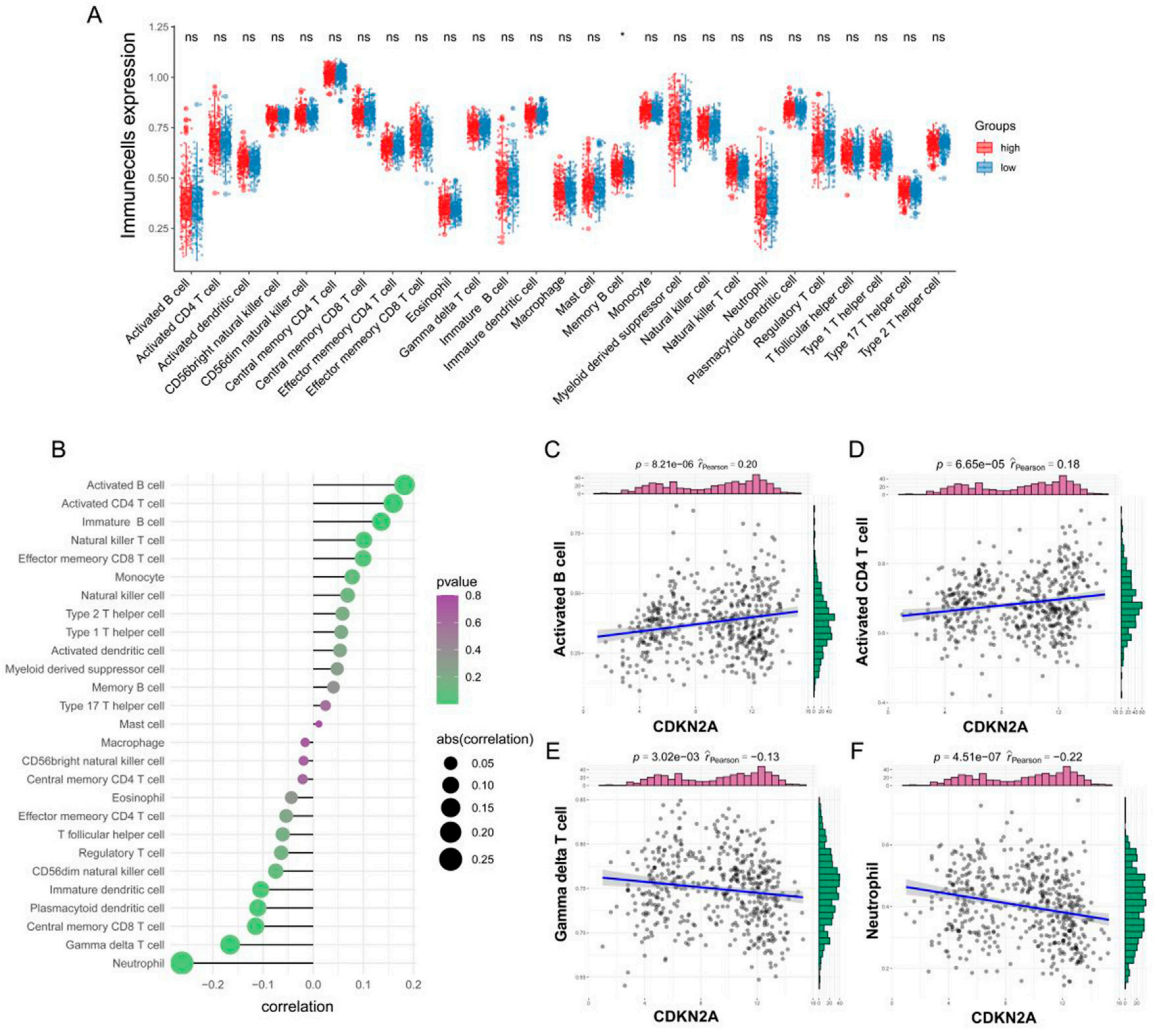
Correlation between CDKN2A and immune cells. **(A)** Differences in immune cell infiltration between the high- and low-CDKN2A-expression groups were examined *via* ssGSEA; **(B)** Correlation between CDKN2A and the infiltration of immune cells; **(C–F)** Correlation plots demonstrating the relationship between the top four immune cell types and CDKN2A.

ssGSEA revealed that the infiltration of memory B cell was higher in the low-CDKN2A-expression group; however, no significant difference was observed in the infiltration of other immune cells between the two groups (Figure 3A and Supplementary Figure S1A). Correlation analysis revealed a co-expression relationship among 28 immune cell types (Supplementary Figure S1B), and the correlation between CDKN2A and immune cells indicated that CDKN2A promoted immune cell infiltration (Figure 3B). CDKN2A was significantly positively correlated with activated B-cell (cor = 0.182, *p* < 0.001) and activated CD4 T-cell (cor = 0.160, *p* < 0.001) (Figures 3C, D) and significantly negatively correlated with neutrophils (cor = −0.262, *p* < 0.001) and gamma-delta T-cell (cor = −0.166, *p* < 0.001) (Figures 3E, F). This suggests that CDKN2A is involved in tumor immune microenvironment homeostasis.

### Gene network and mutation analysis of CDKN2A

**FIGURE 4:**
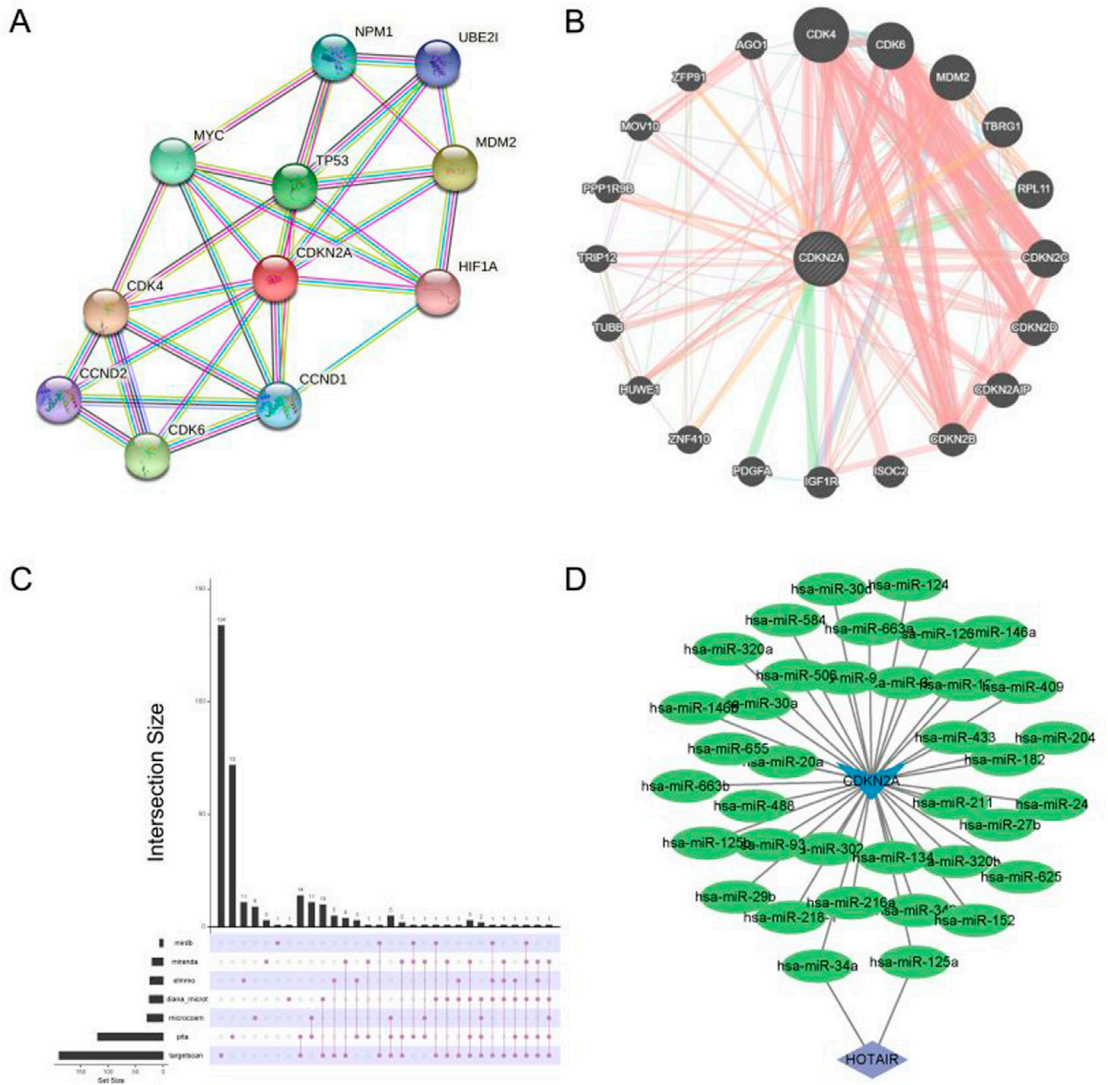
Single gene network diagram. **(A)** PPI network analysis of CDKN2A; **(B)** Gene interaction analysis of CDKN2A using geneMANIA; **(C)** miRNAs targeted by CDKN2A were predicted based on data extracted from seven databases; **(D)** Visualization of the ceRNA network maps using Cytoscape.

A protein–protein interaction (PPI) network is shown in Figure 4A. The gene interaction network of CDKN2A was visualised using geneMANIA (Figure 4B). In the ceRNA network of CDKN2A, HOTAIR was found to be closely associated with has-miR-34a and has-miR-125a (Figures 4C, D). HOTAIR may regulate has-miR-34a and has-miR-125a in HNSCC, thus exerting a regulatory effect on CDKN2A expression (Figure 4D). In addition, CDKN2A had a higher mutation rate at the *p*. R80 locus than at the *p*. R58 and *p*. W110 loci (Supplementary Figure S2).

### Molecular docking results

**FIGURE 5:**
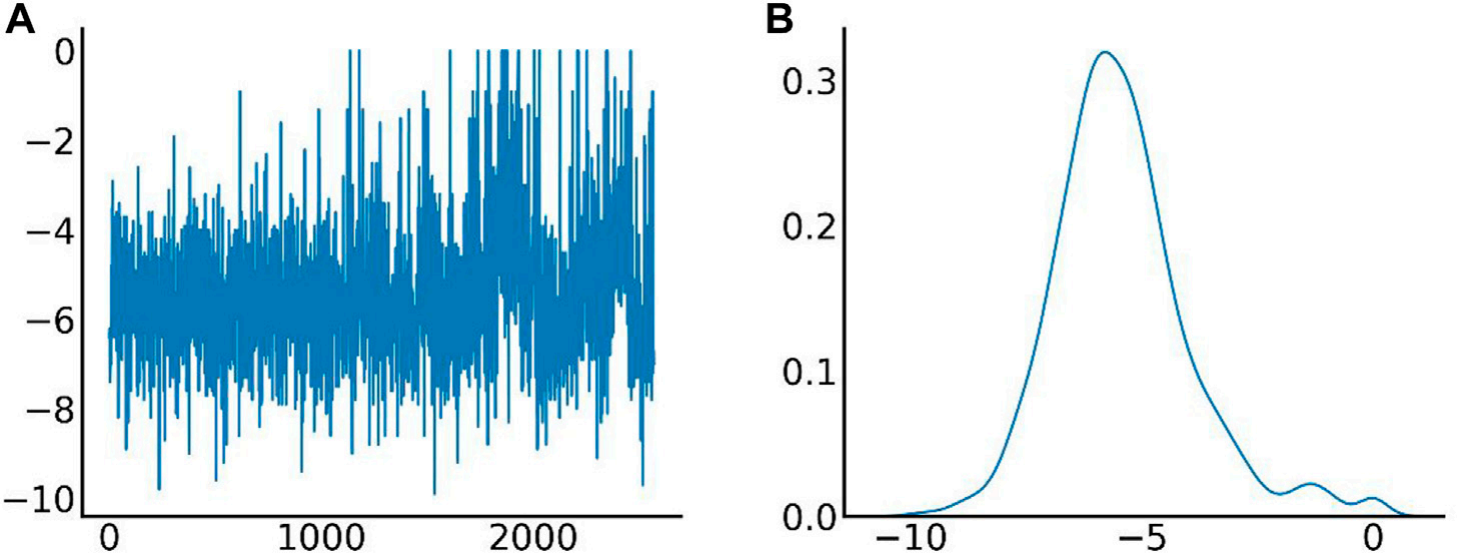
Different amino acid binding modes. **(A)** The X-axis represents the serial number of FDA-approved drugs, and the *Y*-axis represents the docking fraction (unit: kcal/mol); **(B)** Distribution of docking fractions. The horizontal coordinate represents the docking fraction (unit: kcal/mol), and the vertical coordinate represents the distribution probability.

**FIGURE 6:**
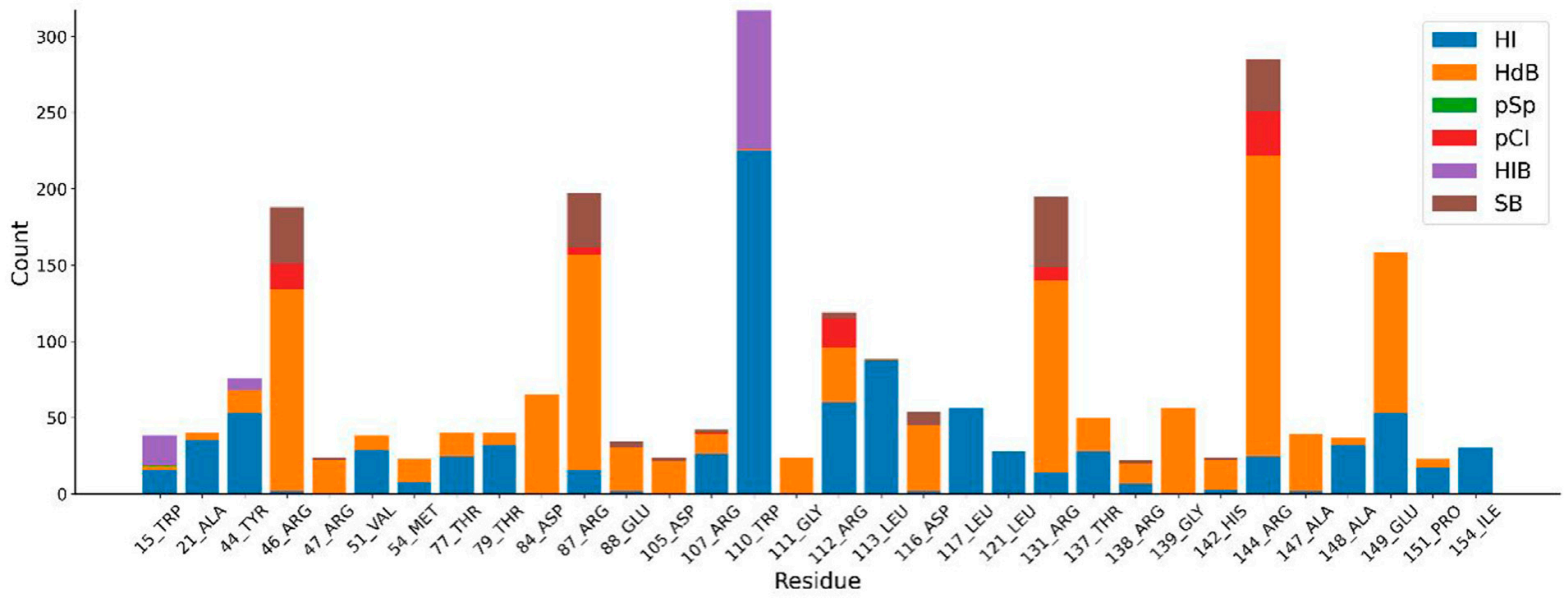
Different amino acid binding modes. Blue represents hydrophobic interactions (HI), orange represents hydrogen bonds (HdB), green represents π-π stacking (pSp), red represents π-cation interactions (pCI), purple represents halogen bonds (HIB) and brown represents salt bridge (SB).

The distribution of docking fractions for virtual screening is shown in Figure 5. The binding affinity of proteins and small molecules can be obtained after molecular docking. The docking fraction for each ordinal number corresponds to −5.42 kcal/mol, with a mean value of −5.42 kcal/mol. Based on a potential screening threshold of −7 kcal/mol, the screening rate is 12.13% (308/2,539), i.e., 12.13% of small molecules have the potential for precursor optimization as Potential dead drug set (PLDS). The mid-docking effects of PLDS were examined to avoid analytical bias caused by transient false-positive docking results. There were 104 residue sites contacted (Supplementary Figure S3). A cut-off of 20 was selected to exclude transient positive contacts. The results are shown in Figure 6. The orange colour represents hydrogen bonds; the number share is the main interaction force [47.41% (1427/3010)] and the amino acid residue contact sites include 46-ARG, 47-ARG, 54-MET, 84-ASP, 87-ARG, 88-GLU, 105-ASP, 111-GLY, 116-ASP, 131-ARG, 138-ARG, 139-GLY, 142-HIS, 144-ARG and 147-ALA. The blue colour represents hydrophobic interactions, which are the main interaction forces [38.27% (1152/3010)], and the amino acid residue contact sites include 21-ALA, 44-TYR, 51-VAL, 77-THR, 79-THR, 107-ARG, 110-TRP, 112-ARG, 113-LEU, 117-LEU, 121-LEU, 137-THR, 148-ALA, 149-GLU, 151-PRO and 154-ILE. Molecular docking analysis suggests that plicamycin binds CDKN2A most strongly.

### Molecular dynamics and cellular effects of plicamycin

**FIGURE 7:**
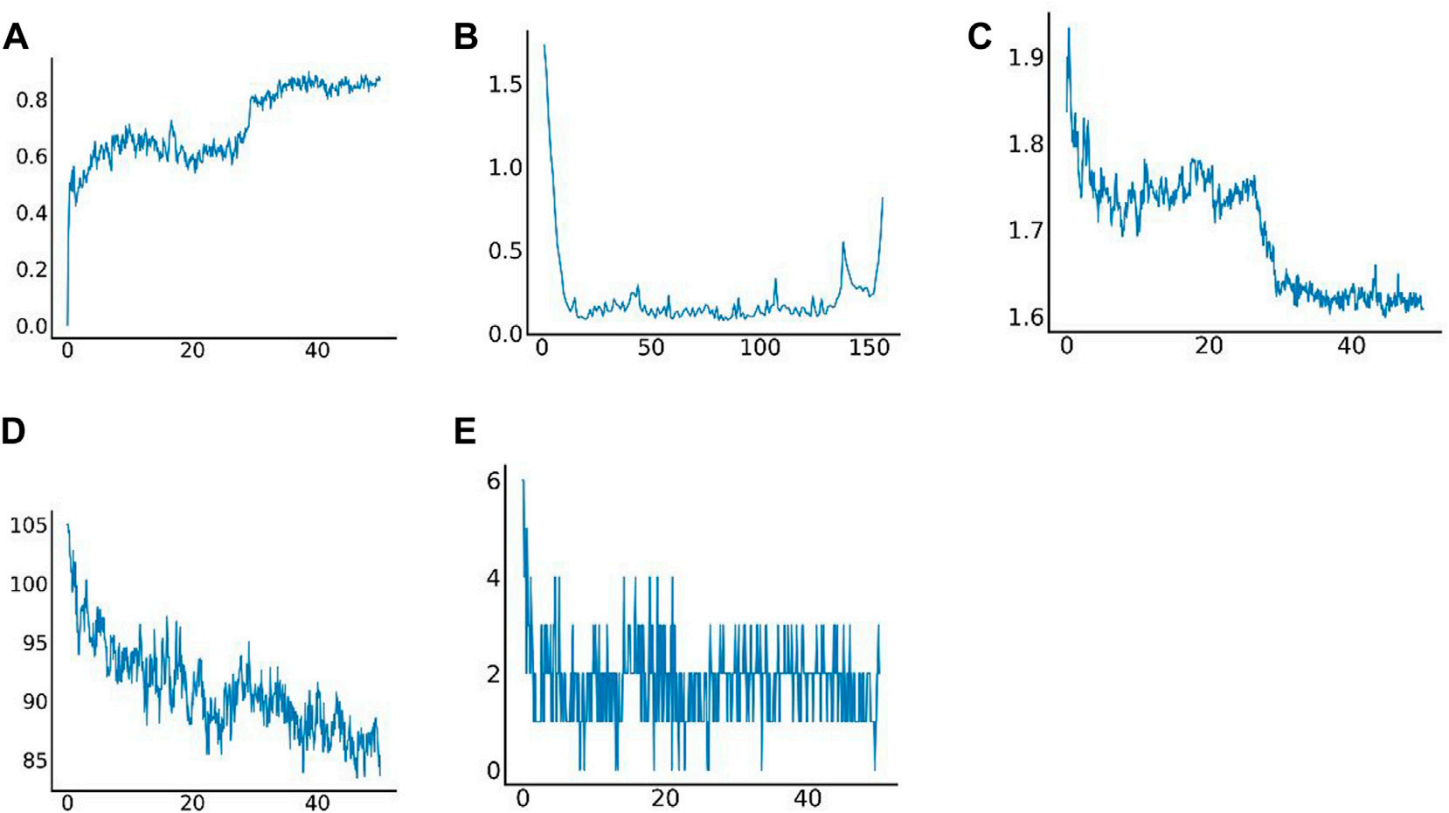
Results of molecular dynamics simulation. **(A)** Root-mean-square deviation (RMSD) of complex MDS; **(B)** Root-mean-square fluctuation (RMSF) of complex MDS; **(C)** Variations in the radius of gyration (Rg) of complex MDS; **(D)** Variations in SASA of the protein in 0–100 ns of complex MDS; **(E)** Variations in hydrogen bonding in the stable complex.

**FIGURE 8:**
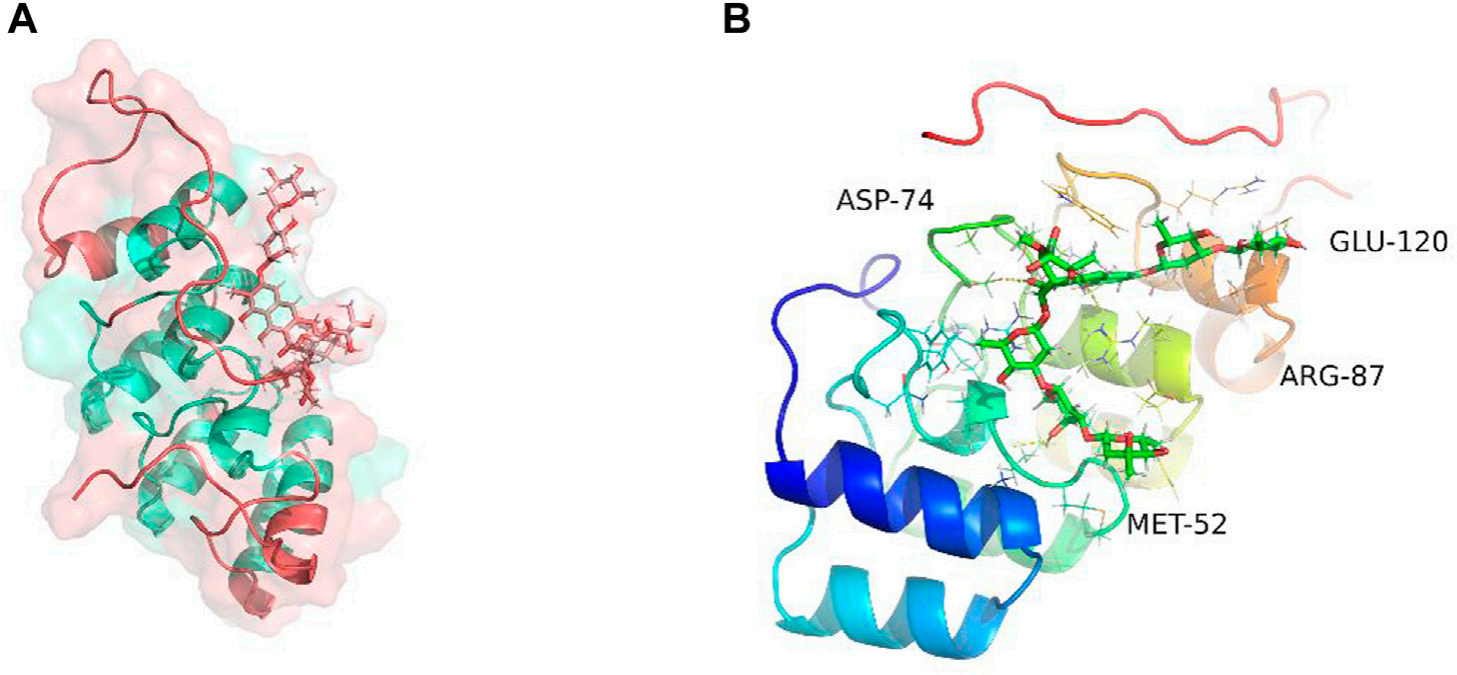
Binding site of plicamycin on the CDKN2A protein. **(A)** Docking pose; **(B)** Amino acids and docking sites.

Molecular dynamics simulation (MDS) is an important method for assessing the stability of complexes in an aqueous solution (Hollingsworth and Dror, 2018; Hildebrand et al., 2019; Huang et al., 2022; Kang et al., 2022; Zhang et al., 2022). Stability of a system can be measured by the atomic root-mean-square deviation (RMSD). In Figure 7A, RMSD values fluctuated between 20 and 30 ns due to transient instability and then remained stable at 30–50 ns. Root-mean-square fluctuations (RMSF) reflect changes in the local sites of the system during MDS. According to Figure 7B, amino acids at positions 1–15, 33–44, 58, 90, 107, and 124–156 fluctuated more than other amino acids. Figure 7C shows that the radius of rotation (Rg) is an important measure of architecture tightness. In Figure 7D, the solvent-accessible surface area (SASA) of the protein decreases steadily over 0–100 ns, indicating favourable binding and progressive protein tightening. Figure 7E shows the potential-energy curve of hydrogen bonded complexes in their steady state. Figures 8A, B show the binding site, docking pose, and overall protein structure.

**FIGURE 9:**
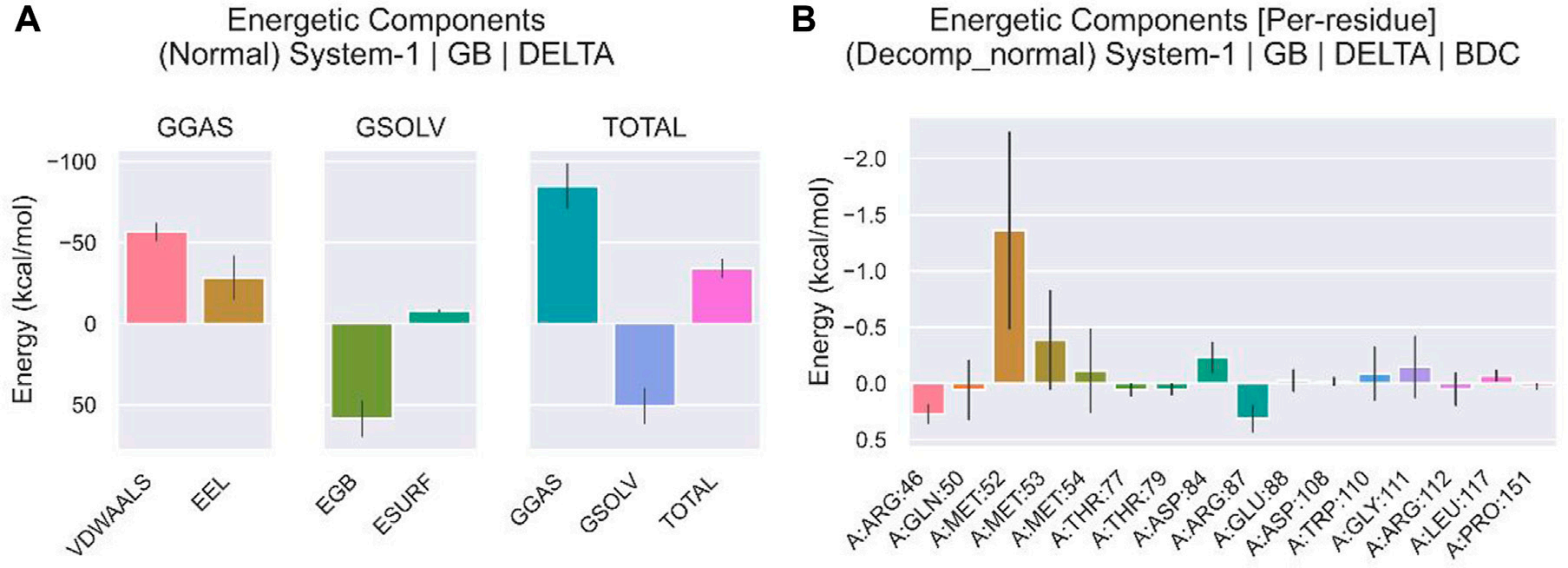
Energy analysis of molecular dynamics simulations. **(A)** Binding free energy of protein–ligand complexes. VDWAALS, van der Waals energy; EEL, electrostatic energy; EGB, polar solvation energy; ESURF, non-polar solvation energy; GGAS, total gas phase free energy; GSOLV, total solvation free energy; G_total_ free energy = GSOLV + GGAS. **(B)** The relationship between each contact residue and the binding energy.

**FIGURE 10:**
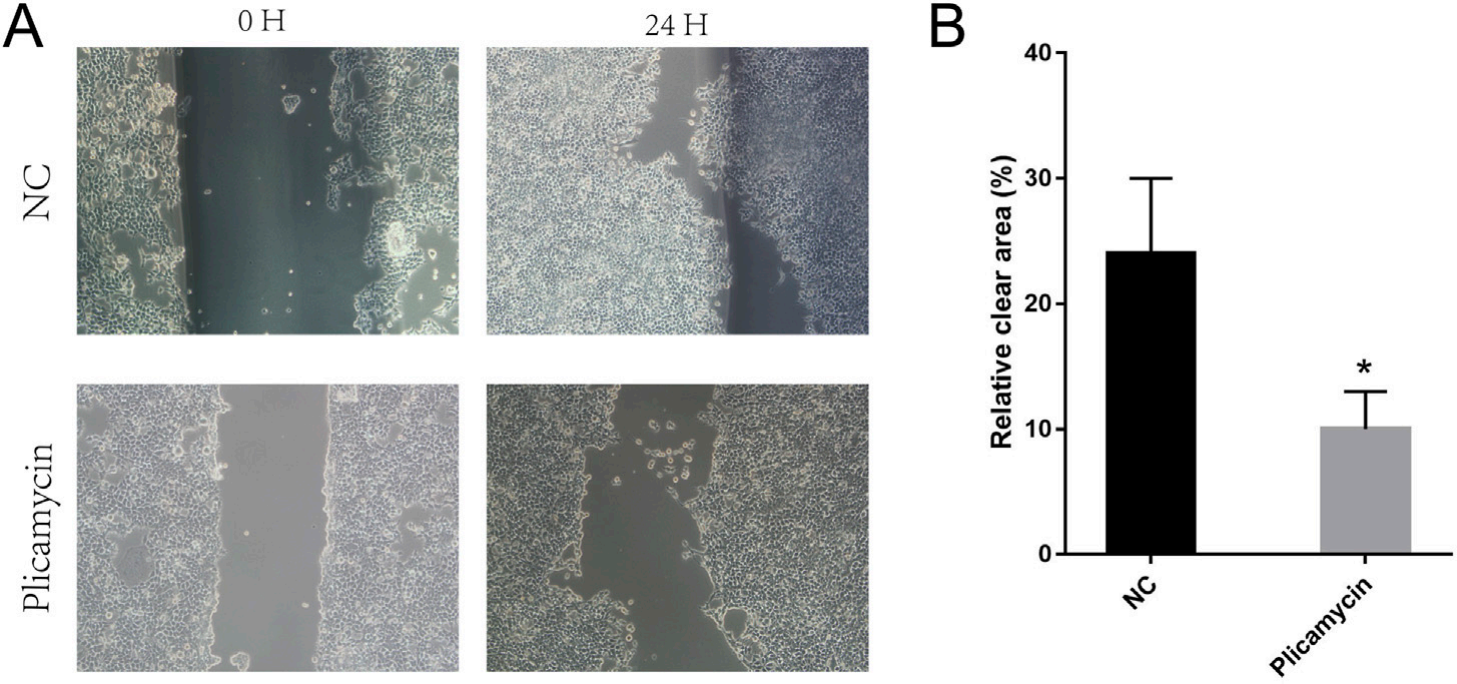
Pplicamycin treatment reduced the mobility of TU212 cells. **(A)** Wound-healing assay for migration activity of untreated (upper panel) and treated (lower panel) TU212 cells. After 24 h, we captured representative images of the migrating cells. **(B)** Quantitative analysis of untreated TU212 invasion effects compared to treated TU212 invasion effects. Invasive cells were counted on average.

We compared the binding free energies of the two solvated molecules in their bound and free states, as well as the binding free energies of various solvated conformations of plicamycin (Figure 9A). Analysis of the variation of the binding free energy with MDS revealed that the total free energy (G_total_) was <0, indicating a likely interaction between the CDKN2A protein and plicamycin. The binding energy of both van der Waals and electrostatic interactions was <0, indicating that hydrophobic interactions and electrostatic energy contribute to the binding between CDKN2A and plicamycin. Non-polar interactions favored binding, whereas polar solvation didn’t. Positive values of polar solvation energy (EGB) indicate that non-polar interactions favored binding. MET-52, MET-53, MET-54, and ASP-84 contact residues of the complex have free energies of zero, indicating they are the major binding sites for CDKN2A and plicamycin (Figure 9B). There was a positive free energy difference between ARG-46 and ARG-87, indicating that plicamycin binds poorly to CDKN2A at these sites. In the stable complex, the contact residue MET-52 contributes to the major binding force (Figure 9B). Furthermore, we found that plicamycin treatment reduced the mobility of TU212 cells in scratch experiments (Figure 10A, B). Accordingly, plicamycin inhibited HNSCC progression in cellular assays. Based on molecular dynamics simulations, this study shows that plicamycin targets CDKN2A to improve patient outcomes.

## Discussion

Cuproptosis, a newly discovered form of cell death, is involved in the accumulation of intracellular copper and possesses the common features of multiple regulated cell death (RCD) mechanisms. Genes associated with cuproptosis play an important role in the prognosis of various tumours such as melanoma and lung cancer. (Shanbhag et al., 2021; Yun et al., 2022). In this study, CDKN2A, a cuproptosis-associated gene, was identified as a novel biological marker for predicting the prognosis of patients with T2-stage HNSCC compared with T3-and T4-stage HNSCC. GSEA and GSVA revealed that CDK2NA overexpression was associated with the cell cycle. Positive correlations were found between CDKN2A expression and activated B and CD4 T cells, but negative correlations were found between CDKN2A expression and neutrophils and gamma-delta T cells. Molecular docking and MDS analysis revealed that plicamycin inhibits HNSCC progression by acting on CDKN2A.

CDKN2A contributes to cell proliferation and angiogenesis by being a member of the INK4 family of tumour suppressor genes (Zhang et al., 2016; Zhao et al., 2016). CDKN2A contains four exons, namely, exons 1α, 1β, 2 and 3. It possesses independent promoters for their respective proteins: exons 1, 2 and 3 encode P16INK4a and exons 1, 2 and 3 encode P14ARF, thus they are both independent proteins (Li et al., 2011). The cyclin-dependent kinase (CDK) inhibitor p16INK4a inhibits the phosphorylation of retinoblastoma protein (pRb) by binding to CDK4 and CDK6. Hypophosphorylated pRb inhibits the transcription factor family E2F, impairing cell cycle progression from G1 to S phase (Rubin, 2013; van den Heuvel and Dyson, 2008). By forming a trimeric complex with MDM2 and p53, P14ARF inhibits the degradation of P53 by MDM2, resulting in G1 and G2 arrest (Pollice et al., 2008). CDKN2A expression is correlated with the development and prognosis of various tumours, including hepatocellular carcinoma, pancreatic cancer and melanoma (Zeng et al., 2018; Kimura et al., 2021; Luo et al., 2021). Copy number deletion in CDKN2A and low expression of p16INK4a indicate a poor prognosis and can be used as independent prognostic predictors in HNSCC (Chen et al., 2018; Cury et al., 2021). A meta-analysis on HNSCC reported that methylation of CDKN2A is significantly correlated with tumorigenesis, progression and metastasis and can be used as a potential diagnostic marker (Zhou et al., 2018). Similar results were obtained in this study, indicating that CDKN2A plays an important role in regulating the tumour cell cycle and may serve as a prognostic marker in HNSCC.

Tumor-infiltrating immune cells are critical components of the tumour microenvironment and are highly predictive of prognosis and treatment outcomes (Nguyen et al., 2016; Guo et al., 2020). In this study, CDKN2A was positively correlated with the infiltration of activated B and CD4 T cells in the tumour microenvironment, leading to a better prognosis of HNSCC. The IGJ gene encodes CD19 and the J-chain gene, typical markers of B cells. In HNSCC, CD19 and J-chain expression were studied, and B cell infiltration in the tumor microenvironment indicated a better prognosis (Kim et al., 2020). Moreover, anti-blockade of PD-1/PD-L1 immune checkpoints in an AT-84-E7 murine model of HNSCC led to tumor enlargement by depleting B cells in the tumor microenvironment by regulating B cell activation and germinal center formation (Kim et al., 2020). This suggests that B cell infiltration and activity play an important role in the treatment of tumors. Hladíková et al., (2019) observed a high abundance of tumor-infiltrating B cells (TIL-Bs) in the HNSCC tumor microenvironment, which may interact with CD8^+^ T cells to promote tumor growth (). They produce tumour-associated antibodies and cytokines, which exert cytotoxic effects on tumours, as well as presenting tumour-associated antigens (TAA) (Candolfi et al., 2011; Mahmoud et al., 2012; Shi et al., 2013; Germain et al., 2014). Infection with the human papillomavirus (HPV) is causing an increase in HNSCC. HNSCC with HPV-positive microenvironment contain more CD4^+^ T cells, which result in a better prognosis and response to radiotherapy than those with HPV-negative microenvironment. However, whether this better prognostic performance is related to CD4^+^ T cell infiltration remains unclear (van Kempen et al., 2016). Multiple studies have shown that neutrophils can promote tumour development and angiogenesis through the secretion of cytokines such as vascular endothelial growth factor, hepatocyte growth factor, IL-6 and IL-8 (McCourt et al., 1999; McCourt et al., 2001; Schaider et al., 2003). Low CDKN2A expression was associated with neutrophil infiltration and poorer prognosis in the present study. Yang et al. reported in a previous study that high neutrophil infiltration can result in the generation of reactive oxygen species, arginase and nitric oxide, causing lymphocytes to be suppressed, T cells to be activated, and, ultimately, tumour growth (Gooden et al., 2011; Yang et al., 2019). Watermann et al., (2021) also found a link between neutrophil infiltration and tumour malignancy in the microenvironment of recurrent HNSCC.

There is a positive correlation between CDKN2A expression and activation of B cells and CD4^+^ T cells. There is evidence that CD8^+^ and CD4^+^ T cells infiltrate the microenvironment to ensure the beneficial effects of gamma-delta T cells on prognosis (Nielsen et al., 2017; Lu et al., 2020). Gamma-delta T cells are important immune cells in the mucosal region responsible for removing pathogens and maintaining the integrity of the epithelium. Lu et al. found a high abundance of gamma-delta T cells infiltration in the tumour microenvironment of patients with HNSCC with a better prognosis. However, Bas et al. examined gamma-delta T cell in the peripheral blood of patients with HNSCC and found that their high abundance was associated with the recurrence of HNSCC (Bas et al., 2006). Therefore, CDKN2A, an immune cell related gene, can aid in improving the prognosis and microenvironment of tumors by inhibiting immune cells that activate B and CD4 T cell, neutrophils and gamma-delta T cells.

Plicamycin, also known as mithramycin A, is a natural polycyclic aromatic polyketide compound that inhibits SP1 transcription factor binding to DNA, which interferes with biological processes like tumour cell proliferation, apoptosis, angiogenesis, invasion, and metastasis (Beishline and Azizkhan-Clifford, 2015; Schweer et al., 2021). Plicamycin is a natural polycyclic aromatic polyketide that inhibits SP1 transcription factor binding to DNA, impeding apoptosis, angiogenesis, invasion, and metastatic processes in cancer cells (Saha et al., 2015). In mouse models of lung cancer, low SP1 expression can effectively inhibit tumour growth and nicotine-induced lung cancer cell growth (Brown et al., 2013). The histone methyltransferase gene SETDB1 is an important gene in the development and metastasis of melanoma *in vivo*. Several studies have shown that plicamycin can effectively target the activity of SP-1 protein on the SETDB1 promoter to inhibit SETDB1 expression, thus offering a beneficial therapeutic strategy for melanoma (Federico et al., 2020). Plicamycin, an inhibitor of SP1, can inhibit tumour progression in several cancer types and has been used for the treatment of lung, breast and gastrointestinal tract cancers in phase II clinical trials, with good efficacy. However, the role and mechanisms of action of plicamycin in HNSCC remain to be elucidated. In this study, strong binding was observed between plicamycin and CDKN2A through molecular docking and MDS, suggesting that plicamycin improves the prognosis of HNSCC by targeting CDKN2A at the molecular level. However, the relationship between CDKN2A and SP1 warrants further investigation. The results of this study are mainly based on bioinformatics analysis and MDS, and they aren’t validated *in vivo* or *in vitro*. Additionally, plicamycin’s effects on HNSCC should be studied in large clinical trials.

## Conclusion

CDKN2A, which is closely related to the maintenance of copper metabolic homeostasis in the body, is a biomarker of HNSCC and may improve its prognosis by regulating the cell cycle and immune cell infiltration. Further, plicamycin targets and binds CDKN2A, offering a novel strategy for the treatment of HNSCC in the future.

## Data Availability

Publicly available datasets were analyzed in this study. This data can be found here: Head and neck squamous cell carcinoma data were obtained from TCGA database (https://portal.gdc.cancer.gov).

## Supplementary material

Supplementary Figure S1 Correlation among immune cell types. **(A)** Heat map demonstrating immune cell infiltration in the high- and low-CDKN2A-expression groups; **(B)** Correlation among different immune cell types.

Supplementary Figure S2 CDKN2A mutation sites.

Supplementary Figure S3 PLDS shows a medium docking effect with a total of 104 residue sites.
