# Supplementary figures and images for: Cuproptosis-associated CDKN2A is targeted by plicamycin to regulate the microenvironment in patients with head and neck squamous cell carcinoma
Source: Front Genet. 2023 Jan 9;13:1036408. doi: 10.3389/fgene.2022.1036408 (PMC9868476; doi:10.3389/fgene.2022.1036408)

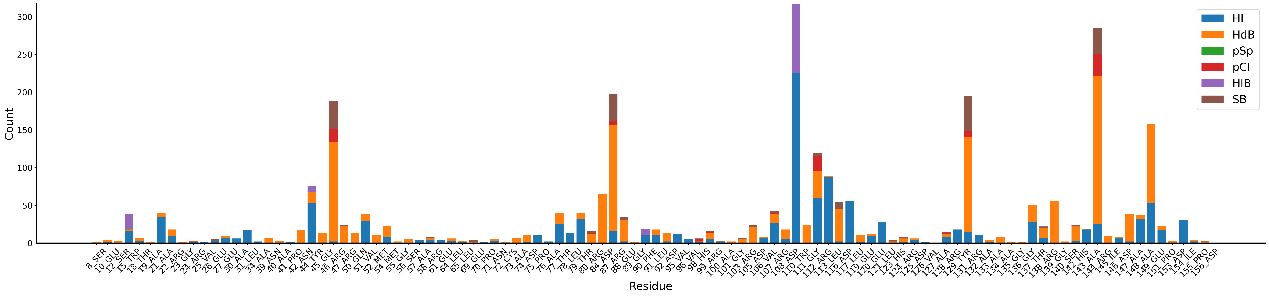

Supplement: Supplementary file 1 [file Image3.JPEG]

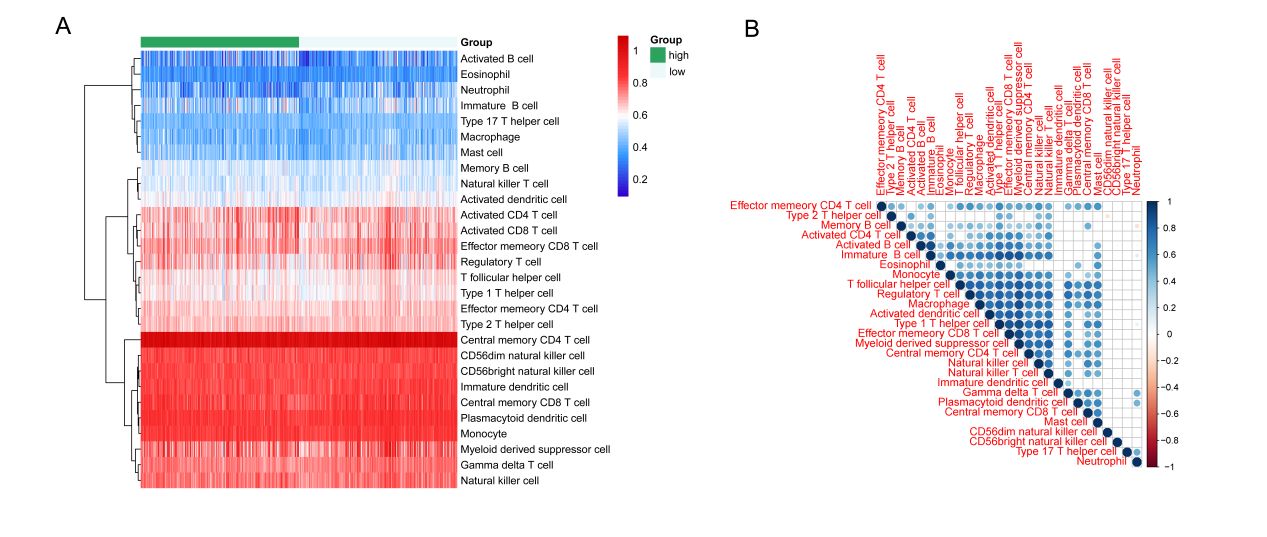

Supplement: Supplementary file 2 [file Image1.JPEG]

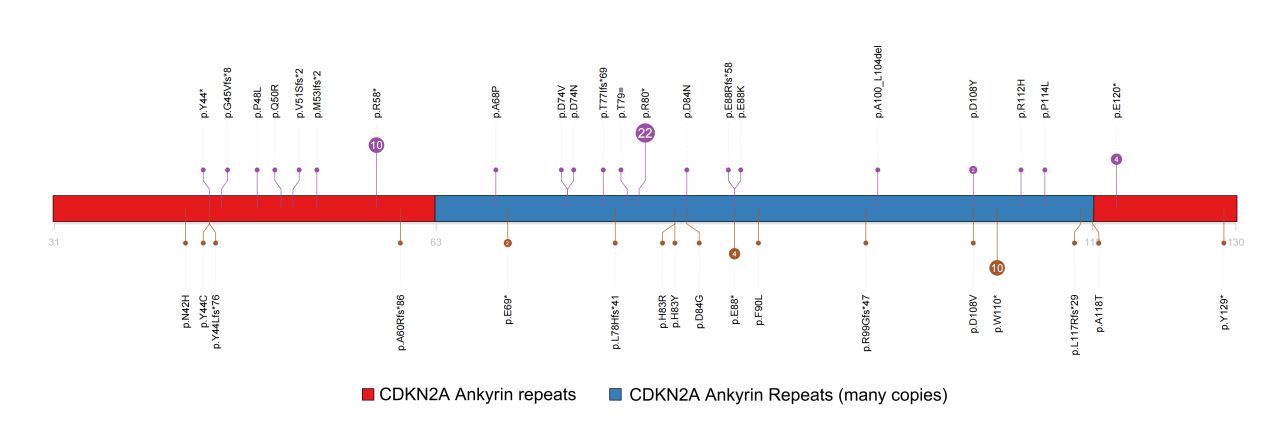

Supplement: Supplementary file 3 [file Image2.JPEG]
